# Coronary artery disease: physiology and prognosis

**DOI:** 10.1093/eurheartj/ehx226

**Published:** 2017-05-26

**Authors:** Thomas J. Ford, David Corcoran, Colin Berry

**Affiliations:** 1 British Heart Foundation Cardiovascular Research Centre, University of Glasgow, UK; 2 University of New South Wales, Sydney, Australia

**This editorial refers to ‘Fractional flow reserve and pressure-bounded coronary flow reserve to predict outcomes in coronary artery disease’^†^, by J.-M. Ahn *et al.*, on page 1980.**

The ‘stenosis centric’ approach to the diagnosis of coronary artery disease (CAD) neglects the broader pathophysiology of angina and disorders of coronary artery function (*Figure 1*). Accordingly, we propose the term ‘stable coronary artery syndrome’ in order to reflect the distinct and related pathologies of focal and diffuse CAD, as well as coronary microvascular and vasospastic disorders, that may reduce myocardial perfusion and provoke ischaemia in individual patients.

**Figure 1:**
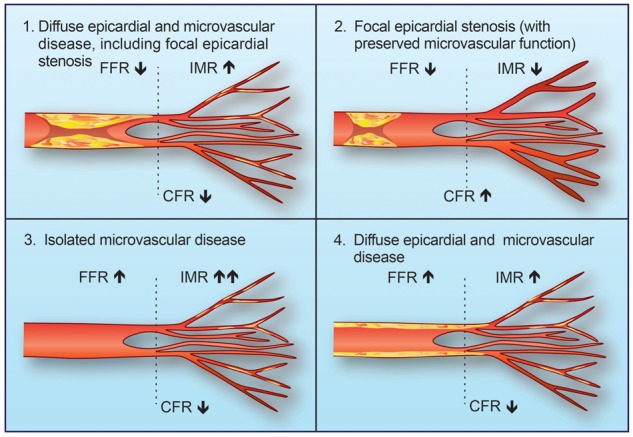
Stable coronary artery syndromes: different combinations of focal, diffuse, and microvascular coronary artery disease (CAD) contribute to myocardial ischaemia. Extreme forms of focal or diffuse coronary disease may result in discordant abnormalities of either fractional flow reserve (FFR) or coronary flow reserve (CFR), respectively. IMR, index of micro-circulatory resistance.

Evidence linking parameters of coronary artery function with prognosis has evolved substantially in the last four decades. Coronary flow reserve (CFR) was first described as the ratio of maximum stress flow to rest flow for a given arterial distribution with or without a stenosis or diffuse narrowing.^1^ CFR determined non-invasively using positron emission tomography (PET) is associated with the risk of major adverse cardiac events (MACE) in the future, independent of clinical variables and the number of ischaemic myocardial segments.^2^^,^^3^ Fractional flow reserve (FFR) was subsequently described as a pressure-derived index to quantify the relative reduction in coronary artery blood flow due to a coronary stenosis as compared with the flow in the same artery in the absence of the stenosis.^4^ Invasively measured CFR (via thermodilution or Doppler wire methods) is reported to be prognostically important even when FFR is preserved. van de Hoef *et al*. demonstrated the potential clinical relevance of discordant CFR and FFR values, showing that in a pooled group of patients with stable coronary artery disease patients with a reduced Doppler-derived CFR (defined as <2.0) but preserved FFR (≥0.75) were at a higher risk of adverse clinical events at 5-year follow-up, compared with patients with concordantly preserved CFR and FFR values [relative risk = 5.0, 95% confidence interval (CI) 2.4–10.2; *P* < 0.001).^5^ Invasively measured CFR and FFR are discordant in ∼40% of patients, which reflects the distinct compartments of the coronary circulation. Johnson *et al*. demonstrated that discordant CFR and FFR values may be explained by fundamental differences in coronary pathophysiology at the extremes of focal and diffuse CAD.^6^ For example, severe focal epicardial coronary disease may be reflected by a reduced FFR (<0.80) and a preserved CFR (>2.0), whereas diffuse coronary plaque without a focal stenosis may be reflected by a preserved FFR (>0.8) and a reduced CFR (<2.0) (*Figure 1*).

In this issue of the journal, Park *et al*. report their analysis of the comparative prognostic utility of FFR and ‘pressure-bounded’ coronary flow reserve (pb-CFR) in a study involving 1837 patients (2088 coronary lesions) enrolled in a multicentre South Korean clinical registry.^7^ pb-CFR is a novel parameter determined by estimating the upper and lower physiological limits of CFR values using resting and hyperaemic pressure data integrated into a mathematical model of pressure and flow. Pb-CFR was shown to have reasonable diagnostic accuracy of 84.4% in a recent validation study based on individual patient haemodynamic data from the DEFER trial.^8^ Patients in the current study were dichotomized into two groups based on a low (<2.0) or high (≥2.0) CFR. A key strength of this analysis is the comparatively large samples size reflecting a prospectively enrolled population of invasively managed patients with a broad range of CAD severities. Further, the pb-CFR values were determined post-hoc; therefore, the data were not available to treating clinicians and so could not have influenced their clinical decisions. The clinical endpoints were adjudicated by a central committee blind to the pb-CFR values. The investigators found that the composite primary endpoint of MACE (cardiac death, myocardial infarction, and repeat revascularization) was predicted by FFR but not by pb-CFR, which had a neutral prognostic implication when adjusted for clinical variables. In a per-lesion analysis, during a median follow-up of 1.9 years (interquartile range: 1.0–3.0 years), the incidence of MACE did not differ between lesions with pb-CFR <2 vs. pb-CFR ≥2 [4.0% vs. 4.0%; adjusted hazard ratio (aHR) = 0.93, 95% CI 0.59–1.48; *P* = 0.76). FFR was predictive of future events, with MACE occurring in 5.7% of lesions with FFR ≤0.80 vs. 2.5% of lesions with FFR >0.80 (aHR = 2.46, 95% CI 1.40–4.31; *P* = 0.002). Incorporation of FFR improved the prediction of MACE (global χ^2^ 38.8–48.1; *P* = 0.002); however, pb-CFR did not confer any additional prognostic utility (48.1–48.2; *P* > 0.99).

The authors should be congratulated for undertaking this large prospective cohort study including the application of mathematical modelling to determine pb-CFR. Nonetheless, the study design is qualified in certain respects. The median follow-up duration of 1.9 years is rather intermediate for a natural history study of coronary atherosclerosis, and the composite primary outcome event rate was predominantly driven by repeat revascularization. CFR is not a test of coronary atherosclerosis and, while impairment of vasodilator reserve would expectedly be associated with atherosclerotic plaque burden and thus CAD progression, the absence of any association between pb-CFR and MACE in this cohort should not be a surprise. In the per-lesion analysis (rather than a more clinically relevant per-patient analysis) investigating the ‘hard’ secondary endpoints of cardiac death or myocardial infarction, there were only 18 events associated with 2088 lesions (event rate 0.0086%) and, importantly, pb-CFR <2.0 was an independent predictor of these important events (1.5% vs. 0.3%; HR 3.77, 95% CI 1.04–13.7; *P* = 0.044).

The authors highlight other limitations including the lack of an absolute CFR value and the inclusion of less than a quarter of all the interrogated lesions (2088 out of 8633), thus potentially introducing a selection bias. The same limitations of thermodilution and Doppler wire-derived CFR apply to pb-CFR, namely its susceptibility to variations in resting haemodynamics. This point is all the more relevant given that >20% of patients in the current cohort had a recent acute coronary syndrome. An increase resting coronary flow, such as with emotional stress, will reduce CFR and so confound any association between CFR and CAD burden.

Lee *et al*. recently described the associations between FFR and microvascular resistance, as revealed by direct invasive measurement of the index of micro-circulatory resistance (IMR) in 313 patients (16% with unstable symptoms).^9^ They found that in patients with preserved FFR, the worst clinical outcomes occurred in patients in the low CFR (<2.0) and high IMR (≥23) group (aHR = 4.914, 95% CI 1.541–15.663; *P* = 0.007), as compared with groups with other CFR/IMR combinations. Unlike FFR, the utility of CFR in clinical practice is limited by the lack of a clearly defined abnormal threshold, and the general influence of both epicardial and microvascular compartments. Ascribing a binary cut-off value for abnormal/normal CFR ignores its continuous stepwise prediction of MACE akin to blood pressure.^10^ FFR remains the evidence-based physiological test for epicardial CAD to inform revascularization decisions in clinical practice. Where appropriate, we advocate direct invasive measurement of microvascular function [with IMR^11^ or the Doppler-derived hyperaemic microvascular resistance (HMR)], in addition to CFR, in order to assess comprehensively the contribution of each coronary compartment to a patient’s symptoms. pb-CFR is currently available and can be generated automatically from invasive pressure data on next-generation haemodynamic consoles (e.g. Coroventis Research AB). Of course, its utility is based on the premise that pb-CFR, or more fundamentally CFR, is a clinically useful parameter to inform revascularization decisions, which remains the subject of much debate. In this regard, more research is needed.

The study by Park *et al*. in this issue fuels the debate regarding whether the clinical utility of coronary revascularization (i.e. the benefit to patients) can be further refined by incorporating adjunctive information using CFR in addition to (or even instead of) FFR in order to improve clinical outcomes.^12^ The DEFINE-FLOW (Distal Evaluation of Functional Performance With Intravascular Sensors to Assess the Narrowing Effect – Combined Pressure and Doppler FLOW Velocity Measurements, clinicaltrials.gov NCT02328820) study is currently examining this question. In DEFINE-FLOW, percutaneous coronary intervention (PCI) is intended to be performed in patients with an epicardial coronary stenosis with concordantly reduced FFR (≤0.8) and CFR (<2.0) values. On the other hand, medical therapy would be considered for a patient with a diseased coronary artery (FFR ≤ 0.80) associated with a normal CFR ≥2.0.

In patients with a known or suspected coronary artery syndrome, as clinicians we are fortunate now to have diagnostic tools that enable a comprehensive assessment of the functional significance of CAD beyond visual interpretation of the angiogram. The remaining challenges include clinical acumen (listening to our patients), education, technology adoption, and importantly, the gaps in clinical evidence to link treatment decisions informed by the use of such tests with improvements in patient wellbeing.

## Funding

Research support from the British Heart Foundation BHF-FS-14-15-30661 to C.B. and D.C.; RE-13-5-30177 to C.B. and T.F.

**Conflict of interest**: the University of Glasgow (employer of C.B.) holds research and consultancy agreements with Abbott/St Jude Medical.
